# NPH Log: Validation of a New Assessment Tool Leading to Earlier Diagnosis of Normal Pressure Hydrocephalus

**DOI:** 10.7759/cureus.659

**Published:** 2016-06-27

**Authors:** Ignacio Jusué-Torres, Jennifer Lu, Jamie Robison, Jamie B Hoffberger, Alicia Hulbert, Abanti Sanyal, Jan Wemmer, Benjamin D Elder, Daniele Rigamonti

**Affiliations:** 1 Department of Neurosurgery, Loyola University Chicago, Stritch School of Medicine, Maywood, Illinois; 2 Department of Neurosurgery, The Johns Hopkins University School of Medicine; 3 Department of Oncology, The Johns Hopkins University School of Medicine; 4 Department of Biostatistics, Johns Hopkins Bloomberg School of Public Health; 5 Department of Radiation Oncology, The Johns Hopkins University School of Medicine

**Keywords:** normal pressure hydrocephalus, early diagnosis, sensitivity, specificity, diagnostic accuracy

## Abstract

Introduction:

Early treatment of normal pressure hydrocephalus (NPH) yields better postoperative outcomes. Our current tests often fail to detect significant changes at early stages. We developed a new scoring system (LP log score) to determine if this tool is more sensitive in detecting clinical differences than current tests.

Material and Methods:

Sixty-two consecutive new patients with suspected idiopathic NPH were studied. Secondary, previously treated and obstructive cases were not included. We collected age, pre- and post-lumbar puncture (LP) Tinetti, Timed Up and Go (TUG) Test, European NPH scale, and LP log scores. The LP log score is recorded at baseline and for seven consecutive days after removing 40 cc of cerebrospinal fluid (CSF) via LP. We studied the diagnostic accuracy of the tests for surgical indication.

Results:

The post-LP log showed improvement in 90% of people with good baseline gait tests and in 93% of people who did not show any pre-LP and post-LP change in gait tests. Sensitivity, specificity, and accuracy to detect intention to treat when positive post-LP improvements were 4%, 100%, and 24%, respectively, for TUG, 21%, 86%, and 34%, respectively, for the Tinetti Mobility Test, 66%, 29%, and 58%, respectively, for Medical College of Virginia (MCV) grade, and 98%, 33%, and 85%, respectively, for LP log score. Pre-LP and post-LP TUG improvement and pre-LP and post-LP Tinetti improvement were not associated with a surgical indication (p > 0.05). LP log improvement was associated with surgical indication odds ratio (OR): 24.5 95% CI (2.4-248.12) (p = 0.007).

Conclusions:

LP log showed better sensitivity, diagnostic accuracy, and association with surgical indication than the current diagnostic approach. An LP log may be useful detecting NPH patients at earlier stages and, therefore, yield better surgical outcomes.

## Introduction

In 1965, Solomon Hakim described normal pressure hydrocephalus (NPH) as “a mild impairment of memory, slowness, and paucity of thought and action, unsteadiness of gait, and unwitting urination” [1-2]. Hakim’s triad, considered pathognomic of NPH, refers to the contemporaneous presence of gait, cognition, and urinary difficulties in the context of ventriculomegaly [1-2]. Many neurologists and neurosurgeons, therefore, consider the diagnosis of iNPH only in the presence of the three symptoms.

In reality, many patients that benefited from surgery for hydrocephalus do not present with the full “triad.” The complete syndrome may correspond to a late phase of this condition [3-6], as asymptomatic ventriculomegaly observed on magnetic resonance imaging (MRI) may precede the development of the characteristic symptomatology by several years [7]. Furthermore, patients treated for iNPH early after the onset of their symptoms have better postoperative outcomes [6, 8]. The major difficulty is that our current tests often fail to detect significant changes at early stages. The European iNPH Multicentre Study showed that while the resistance to CSF outflow and the CSF tap test have high positive predictive values (PPV > 90%), they cannot exclude patients for surgery, given their very low negative predictive values (NPV < 20%) [9]. Therefore, more sensitive tests are clearly needed to detect iNPH at earlier stages.

To achieve more sensitive testing, an iNPH assessment (LP log) was developed to quantitatively measure this subjective performance deterioration. The objective of this study was to validate this tool and demonstrate as a “proof of concept” that is more sensitive in detecting clinical differences than the currently available testing modalities.

## Materials and methods

Following approval from the Institutional Review Board (NA_00044584), a retrospective review of prospectively collected data was performed. Patient and caregiver consents were obtained before enrollment to the study. The records of all new patients referred to the Hydrocephalus Clinic at a single-institution for evaluation of suspected iNPH from 2013 to 2015 were recorded. 

Inclusion criteria comprised age greater than 60 years, clinical symptoms suggestive of non-previously treated idiopathic hydrocephalus, Evans Index greater than 0.3, and normal morphology of the third ventricle. Patients with a known cause of hydrocephalus, such as trauma, tumor, infection, or bleeding, were excluded. Other exclusion criteria were patients with obstructive hydrocephalus or evidence of bowing of the third ventricular floor and/or the anterior wall of the third ventricle [10] or if patients were previously treated for iNPH with shunting or endoscopic third ventriculostomy.

Demographic factors and clinical information were prospectively collected. Demographic factors included: age, gender, race, and body mass index (BMI). Clinical information included the presence of gait/balance impairment, urinary urgency/incontinence, memory/attention deficits, headaches/dizziness, Hakim’s Triad, and duration of symptoms at presentation. Forty cc of CSF were removed via lumbar puncture (LP) for evaluation of suspected hydrocephalus in all patients. Pre-LP and post-LP functional status were assessed using the Timed Up and Go (TUG) test, Tinetti score, Medical College of Virginia (MCV) gait grade (1 to 6), Mini-Mental State Examination (MMSE), and European iNPH Multicentre Study [5]. TUG and MCV lower values suggest better gait performance. For Tinetti score, MMSE, and European Hydrocephalus Scale, higher values reflect better status.

In the clinic, before performing the LP, the patients and caregivers were routinely asked to measure the patient’s baseline performance status using questions in the new LP log assessment form, as shown in Tables 1-2.

**Table 1:** Patient´s Baseline – Normal Pressure Hydrocephalus Log

	Strongly Agree	Agree	Unsure	Disagree	Strongly Disagree
1. I feel balanced.					
2. I feel confident walking inside and outside.					
3. I am able to stand up and sit down with ease.					
4. I am able to walk up and down stairs and or hills with ease.					
5. I have energy each day to complete my daily tasks.					
6. I am easily able to make plans, problem solve, and move from one task to the next.					
7. I am easily able to pay close and continuous attention to tasks.					
8. I have the motivation to perform daily chores, errands, and call or see my family and friends.					
9. I enjoy listening to music.					
10. I have issues with my urinary urgency.					
11. In the past 3 months, I feel that I can process questions/commands/requests that are made to me, and react appropriately to them without delay or needing of repetition					

**Table 2:** Caregiver´s Baseline – Normal Pressure Hydrocephalus Log

	Strongly Agree	Agree	Unsure	Disagree	Strongly Disagree
1. The patient is balanced.					
2. The patient is confident walking inside and outside.					
3. The patient is able to stand up and sit down with ease.					
4. The patient is able to walk up and down stairs and or hills with ease.					
5. The patient has energy each day to complete their daily tasks.					
6. The patient is easily able to problem solve and move from one task to the next.					
7. The patient is easily able to pay close and continuous attention to tasks.					
8. The patient has the motivation to do daily chores, errands, call or see their family and friends.					
9. The patient enjoys listening to music.					
10. The patient has issues with their urinary urgency.					
11. In the past 3 months, the patient seems to be able to process questions/commands/requests made to them, and can react appropriately with no delay or needing of repetition					

Following the high-volume LP, the patients and caregivers measured the patient’s change in performance using the same questions used for the baseline assessment for seven consecutive days, as shown in Tables 3-4. The patients and caregivers were asked to state if they “strongly agreed, agreed, were unsure, disagreed, or strongly disagreed” with 11 statements describing patient's function regarding activities of daily living.

**Table 3:** Patient’s Post-Lumbar Puncture – Normal Pressure Hydrocephalus 7 Day Log

	Day 1	Day 2	Day 3	Day 4	Day 5	Day 6	Day 7
1. I am more balanced.							
2. I am more confident walking inside and outside.							
3. I am better able to stand up and sit down.							
4. I am better able to walk up and down stairs and hills.							
5. I have more energy to complete my daily tasks.							
6. I am better able to make plans, problem solve, and move from one task to the next.							
7. I am better able to pay close and continuous attention to tasks.							
8. I have more motivation to do daily chores and errands, call or see friends and family.							
9. I enjoyed listening to music more.							
10. I have fewer issues with my urinary urgency.							
11. I feel that I can better process questions/commands/requests made to me, and can react appropriately with no delay or needing of repetition.							

**Table 4:** Caregiver Post-Lumbar Puncture – Normal Pressure Hydrocephalus 7 Day Log

	Day 1	Day 2	Day 3	Day 4	Day 5	Day 6	Day 7
1. The patient is more balanced.							
2. The patient is more confident walking inside and outside.							
3. The patient is better able to stand up and sit down.							
4. The patient is better able to walk up and down stairs and hills.							
5. The patient has more energy to complete their daily tasks.							
6. The patient is better able to make plans, problem solve, and move from one task to the next.							
7. The patient is better able to pay close and continuous attention to tasks.							
8. The patient has more motivation to do daily chores and errands, call or see friends and family.							
9. The patient enjoyed listening to music more.							
10. The patient has fewer issues with their urinary urgency.							
11. The patient can better process questions/commands/requests made to them, and react appropriately with no delay or needing of repetition							

The principal outcome measures were the intention to treat with surgical shunt placement and shunt response. Intention to treat was expressed by the senior author after assessing the clinical and radiological presentation together with the results of the standard testing and the results of the new LP tool. Shunt response was defined using the same criteria as the European iNPH Multicentre Study [9, 11]. Shunt response was recorded as the existence of improvement of at least five points in the European NPH scale between the presentation and the postoperative scores, respectively.

Categorical data was summarized using frequencies and percentages. Continuous data was summarized using median and interquartile range (IQR). Paired Wilcoxon’s rank-sum test was used to assess differences before and after LP for continuous variables and Fisher’s exact test was used for categorical variables. Logistic regression analysis was used to assess if there was a correlation between the studied scales and intention to treat as well as between the scales and shunt response. All reported p-values were two-sided and statistical significance was set at p < 0.05. All analyses were performed using SAS version 9.3 (SAS Institute, Inc., Cary, NC, USA).

## Results

### At presentation

Sixty-two patients were identified following application of the inclusion/exclusion criteria. Patient characteristics are shown in Table 5.

**Table 5:** Patient Demographics Abbreviation: interquartile range: IQR; body mass index: BMI; Timed Up and Go: TUG; Medical College of Virginia gait grade: MCV; Mini–mental state examination: MMSE; intracranial pressure: ICP.

Patient Characteristics at Presentation	Patients (N=62)	
Age at Presentation, years (IQR)	76 (71-80)	
Sex, Female, n (%)	26 (42%)	
Race, n (%)		
Caucasian	59 (95%)	
African-American	2 (3%)	
Other	1 (2%)	
BMI, kg/m^2^ (IQR)	27.6 (23.9-31.5)	
Symptoms/Signs at Presentation, n (%)		
Gait/Balance Impairment	61 (98%)	
Urinary Urgency/Incontinence	54 (87%)	
Memory/Attention Deficits	48 (77%)	
Headaches/Dizziness	17 (27%)	
Presence of Complete Triad, n (%)	41 (66%)	
Duration of Symptoms, months (IQR)	24 (16-48)	
Physical Exam Scoring at Presentation	Pre-LP	Post-LP
TUG*, *seconds (IQR)	15 (12-26)	14 (12-20)
Tinetti*, *median (IQR)	22 (17-25)	24 (20-26)
MCV Scale, median grade (IQR)	3 (2-4)	2 (2-3)
MMSE at presentation*, *median (IQR)	27 (21-29)	
European Hydrocephalus Scale at presentation*, *points (IQR)	52 (30-76)	
Evans Index at presentation, median (IQR)	0.36 (0.34-0.37)	
Opening ICP, cmH_2_O (IQR)	15 (13-19)	

Median age was 76 years. The complete Hakim’s triad was present in 66% of cases. Examining the signs and symptoms separately, 98% of patients presented with gait or balance deterioration, 87% with urinary urgency or incontinence, and 77% with memory or attention deficits. The median Evans index was 0.36; median opening pressure was 15 cm H_2_O, and the median duration of symptoms was 24 months. At presentation, median TUG was 15 minutes; median Tinetti score was 22; median MCV grade was 3, and median European scale score was 52.

### Post LP changes

TUG (p < 0.008), Tinetti (p < 0.001), and MCV grade (p = 0.01) significantly improved following LP. Overall, 57 (92%) patients demonstrated post-LP log improvement. The post-LP log score improvement was statistically significant for both patients (p < 0.001) and caregivers (p < 0.001) independently and combined (p < 0.001).

**Figure 1:**
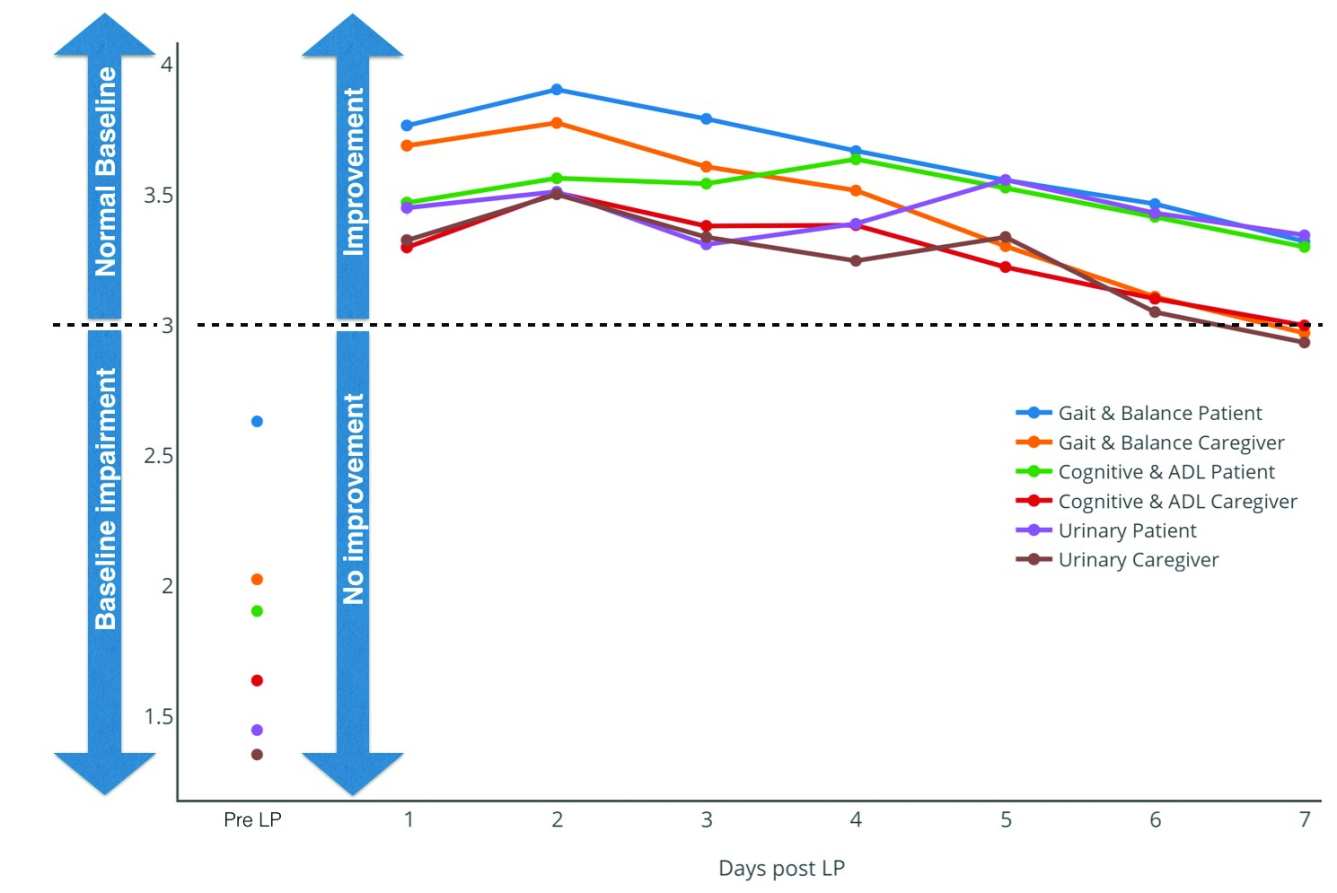
Baseline and post-LP log scores over time comparing the gait/balance, cognitive/ADL, and urinary domains of questions in patients and caregivers. Abbreviations: Activities of daily living: ADL

Interestingly, as shown in Figure 1, the responses to the LP log questionnaire showed a clear initial post-LP improvement with a linear slow return to the pre-LP status.

Patients responded with significantly better performance than caregivers for all questions at both baseline and each day post-LP. The peak improvement, as assessed by the caregivers, was identified on the second-day post-LP for gait, urinary, and cognitive questions. However, the optimal temporal performance for patients in self-assessment was the second-day post-LP for gait, the fourth-day post-LP for cognition, and the fifth-day post-LP for urinary symptomatology.

Patients with significant post-LP log improvement had statistically significant improvement between pre- and post-LP TUG (mean difference: 2.88 seconds, p = 0.01) and Tinetti (mean difference: 2 points, p < 0.0001). Neither TUG nor Tinetti demonstrated any statistical differences between pre- and post-LP timing in patients that did not have post-LP log improvement.

**Figure 2:**
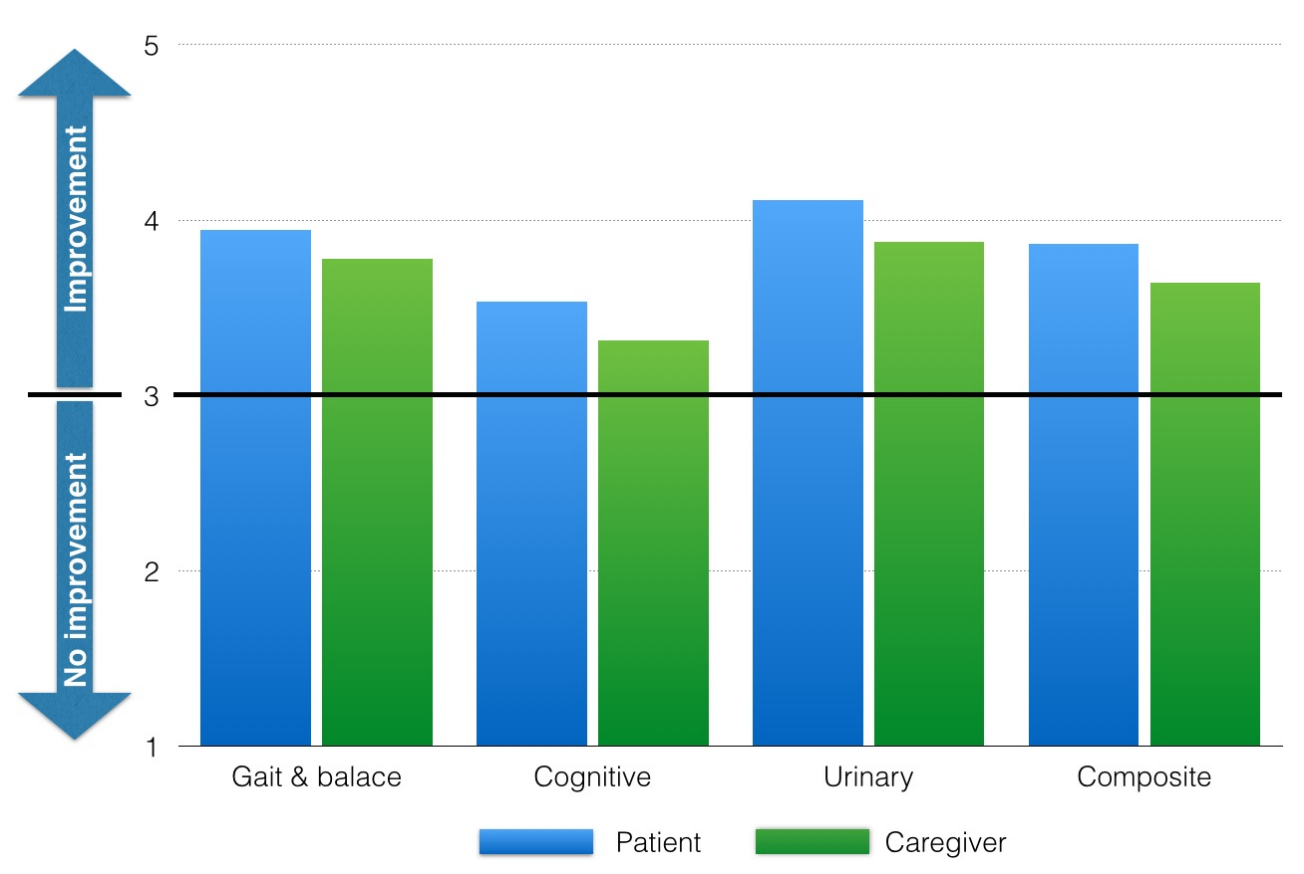
Post-LP log scores of patients with good baseline TUG, Tinetti, and MCV tests.

Patients that had a good baseline gait scoring test (TUG < = 12 seconds, Tinetti > = 25, and MCV grade < = 3) did not show significant statistical change of their post-LP TUG (p = 0.41), post-LP Tinetti (p = 1), or post-LP MCV (p = 0.9). However, 90% of patients with good baseline gait scoring improved in their post-LP log. Figure 2 depicts the mean post-LP log scores in patients with good baseline gait performance.

**Figure 3:**
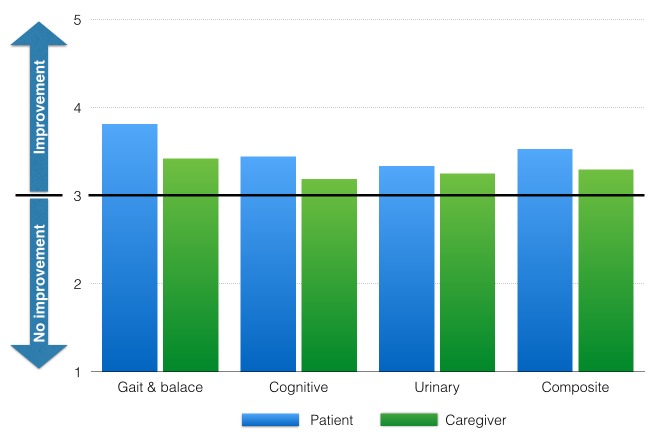
Post-LP log scores of patients without improvement in post-LP TUG, Tinetti, and MCV tests.

The LP log detected a post-LP improvement in all patients that did not have an evident change in their post-LP (TUG difference post-LP to pre-LP < 1 second, Tinetti difference post-LP to pre-LP < 2 points, and MCV grade difference < 1). Figure 3 details the mean post-LP log scores in patients that did not have an evident change in their gait performance post-LP.

### Intention to treat

Surgery was indicated in 53 (85%) patients. Improvement of at least one second in pre-LP to post-LP TUG (p > 0.05) and at least one point in pre-LP to post-LP Tinetti was not associated with a surgical indication (p > 0.05). Post-LP log improvement was significantly associated with surgical indication OR: 24.5 95%CI (2.4­ - 248.12), p = 0.0068), as shown in Table 6.

**Table 6:** Univariate Logistic Regression Analysis for Intention to Treat Abbreviations: Timed Up and Go: TUG; Medical College of Virginia gait grade: MCV; Odds Ratio: OR; Confidence Interval: CI

Post-LP Improvement	OR	95% CI	*p*-value
TUG Improvement	1.68	0.45 - 7.10	0.451
Tinetti Improvement	1.72	0.44 - 6.57	0.42
MCV Improvement	1.99	0.44 - 14.067	0.41
LP Log Improvement	24.50	2.4­ - 248.12	0.0068

Table 7 demonstrates the sensitivity, specificity, and diagnostic accuracy for intention to treat in the TUG, Tinetti, MCV, and LP Log responses. The assessment with the greatest sensitivity and diagnostic accuracy for indication to treat was the LP log.

**Table 7:** Sensitivity and Specificity of the Different Scales for Intention to Treat and Shunt Response Abbreviations: Timed Up and Go: TUG; Medical College of Virginia gait grade: MCV

Intention to Treat	Sensitivity	Specificity	Accuracy
TUG	4%	100%	24%
Tinetti	21%	86%	34%
MCV	66%	29%	58%
LP Log	98%	33%	85%
Shunt Response	Sensitivity	Specificity	Accuracy
TUG	0%	78%	19%
Tinetti	19%	89%	36%
MCV	56%	22%	47%
LP Log	96%	0%	72%

### Surgical outcomes

Seventy-nine percent of shunted patients showed shunt response at their last follow-up (defined as at least five points of post-surgical improvement on the European NPH scale). Pre- and post-LP TUG, Tinetti, or MCV changes were not associated with shunt response (p > 0.05). Association with post-surgical improvement could not be assessed due to a lack of patients that underwent surgery with a lack of improvement in the post-LP log, resulting in an LP log specificity of 0 (Table 3). However, the assessment with the greatest sensitivity and diagnostic accuracy for shunt response was the LP log. 

## Discussion

This study demonstrates how the newly described LP log can detect post-LP differences in patient function when traditional assessments, such as TUG, Tinetti, or MCV fail to do so. The LP log demonstrated better sensitivity, diagnostic accuracy, and association with surgical indication than the currently utilized diagnostic approaches, thus, validating use of the log to aid in the diagnosis of iNPH.

The objective diagnosis of iNPH is complicated by several factors. Idiopathic NPH takes several years to develop and the initial symptoms are soft and easy to miss [4, 12]. Patients with iNPH span a continuum from very healthy and functional to very sick and disabled. MRI scans have helped in the early recognition of ventriculomegaly, often preceding the appearance of any symptoms. At the other end of the spectrum, in the elderly population, comorbidities are much more pronounced and may mask iNPH symptomatology, especially if there is a slow progression [13-15]. 

It is crucial to have the ability to detect changes at earlier timepoint, as there are better outcomes with early treatment [6]. However, our currently used assessments lack sensitivity and often fail to detect significant changes at early phases. In our experience, an initial negative work-up often becomes positive following reassessment at a later time when symptoms have progressed. Deficits that were initially minor then became “measurable” with our tests, proving that our present diagnostic tools are lacking in the early phase and, therefore, treatment is delayed.

The situation may be even more dire in highly functional patients (athletic individuals or those with high IQ) with hydrocephalus. These patients face a greater hurdle, as even a significant decline in their “baseline” function often still falls in the “normal” range, because tests results are reported in comparison to a control population whose baseline level of function is often at a much lower level than the baseline function of these high-functioning individuals. This situation would therefore delay the detection of the early symptoms of iNPH for several years, often resulting in a drastic reduction in function before diagnosis.

Another issue is that current assessments are based on physical exams performed in a clinical environment. Family members and caregivers often report that patients are significantly improved clinic, compared to their daily life, which may be due to several factors. For instance, patients often try to demonstrate optimal performance to the healthcare team while being evaluated. Additionally, the clinic environment does not provide the same challenges as daily life. Therefore, to be relevant, iNPH diagnostic testing needs to be sensitive to the individual baseline functional status and needs to be sensitive enough to measure a variation from baseline. The new LP log compares the changes observed in the daily life of patients after CSF tapping for seven consecutive days, thus, providing longer-term evaluation of patient function following a tap test.

Interestingly, our results showed that the post-LP improvement peak was not the first day post-CSF tapping. Instead, the peak of improvement was most commonly reported by the caregiver on the second day, while the optimal performance for patients was the second day for gait, the fourth day for cognition, and the fifth day for urinary evaluation. This information may explain why differences may not be detected when measuring gait performance on the first day post-LP. This may also explain why it is possible to detect differences in other patients following several days of external lumbar drainage, although this requires hospital admission for several days with increased costs and an increased risk of patient morbidity. This information again highlights the critical importance of longer-term evaluation of patient function following a tap test, which would be challenging in an entirely clinic-based assessment. 

## Conclusions

The LP log showed better sensitivity, diagnostic accuracy, and association with surgical indication than the current diagnostic approach. The LP log may be useful detecting NPH patients at earlier stages and, therefore, yielding better surgical outcomes. Additionally, as it is a patient and caregiver-based assessment, there is limited additional healthcare expenditure.
